# The clinical trajectory of emerging bipolar disorder among the high-risk offspring of bipolar parents: current understanding and future considerations

**DOI:** 10.1186/s40345-017-0106-4

**Published:** 2017-11-22

**Authors:** A. Duffy, C. Vandeleur, N. Heffer, M. Preisig

**Affiliations:** 1 Student Wellness Centre, Department of Psychiatry, Queen’s University, 146 Stuart Street, Kingston, ON K7L3N6 Canada; 2 Department of Psychiatry Lausanne, University Hospital of Lausanne, Lausanne, Switzerland; 3 Medical Sciences Division, University of Oxford, Oxford, UK

**Keywords:** Bipolar disorder, Early course, Antecedents, Stages, Illness trajectory, High-risk, Offspring

## Abstract

**Background:**

Relatively little is known about the onset of bipolar disorder, yet the early illness course is already associated with significant morbidity and mortality. Therefore, characterizing the bipolar illness trajectory is key to risk prediction and early intervention advancement.

**Main body:**

In this narrative review, we discuss key findings from prospective longitudinal studies of the high-risk offspring of bipolar parents and related meta-analyses that inform us about the clinical trajectory of emerging bipolar disorder. Challenges such as phenotypic and etiologic heterogeneity and the non-specificity of early symptoms and syndromes are highlighted. Implications of the findings for both research and clinical practice are discussed.

**Conclusion:**

Bipolar disorder in young people at familial risk does not typically onset with a hypomanic or manic episode. Rather the first activated episode is often preceded by years of impairing psychopathological states that vary over development and across emerging bipolar subtype. Taking heterogeneity into account and adopting a more comprehensive approach to diagnosis seems necessary to advance earlier identification and our understanding of the onset of bipolar disorder.

## Background

Bipolar disorder is a heterogeneous and genetically complex illness (McGuffin et al. 2010). Currently, the diagnosis is based on the manifestation of at least one manic (bipolar I) or hypomanic and depressive episode (bipolar-II). However, prospective studies provide substantial evidence that the first activated (manic or hypomanic) episode is often preceded by one or several depressive episodes typically in adolescence, and not uncommonly by much earlier childhood clinical antecedents (Duffy et al. 2014; Egeland et al. 2012; Mesman et al. 2013). Further, it is estimated to take over a decade from the onset of the first activated episode to recognition of the bipolar diagnosis, and much longer still from the onset of impairing symptoms (Egeland et al. 2000; Judd and Akiskal 2003). This diagnostic delay is associated with devastating consequences including inappropriate treatment, increased hospitalization, medical comorbidity, treatment refractoriness, addiction, school drop-out, underemployment, and suicide (Judd et al. 2005; Keck et al. 2008).

Therefore, earlier identification of emergent bipolar disorder and advances in understanding the nature of illness onset are key research priorities. An important strategic aim to attain these goals is mapping risk processes and markers of illness activity over the trajectory of illness development—from well but at risk, to full-blown illness. An informative approach to accomplish this aim is to prospectively study the high-risk offspring of bipolar parents from childhood over the peak risk period for illness onset. (i.e., adolescence and early adulthood). This design allows for parallel, real-time assessment of risk factors and potential confounds—allowing for the separation of trait from state, critical to the task of identifying risk markers and endophenotypes. Ultimately, this advance will inform the development and timing of specific early interventions; thereby reducing the proportion of individuals reaching end-stage illness and developing a progressive illness course.

## Methods

We completed a selective narrative review of published studies that inform us about the emerging clinical trajectory of bipolar disorder. Included studies (listed in Table 1) had to be prospective and longitudinal in design, include the offspring of at least one parent with bipolar disorder, and report on clinical outcomes at the syndrome level. The purpose of this narrative review is, therefore, to summarize the available evidence informing the clinical picture of emerging bipolar disorder and highlight the importance of heterogeneity in this work. We also discuss how risk factors such as family history can be used to differentiate the early bipolar illness trajectory from otherwise non-specific clinical presentations. Finally, we discuss the clinical and research implications of these findings, highlighting knowledge gaps and future challenges.

**Table 1:** An overview of prospective bipolar offspring studies: study characteristics and psychopathology outcomes

Published work	Cohort characteristics					Parental characteristics				Offspring characteristics				
	Country of origin	Type of cohort	Years of follow-up	Follow-up interval (# interviews)	Drop-out rate (%)	*n*	BD index parent	BD mother (%)	Recruitment	*n*	Mean age at last assessment (range)	Diagnostic interview	Most recent offspring diagnoses % (controls)	Early course of BD
Duffy et al. (1998, 2014)	Canada	Dynamic^a^	Up to 16 (mean 6.3)	Annually	9	113	45% BDI 43% BDII 12% RMDD^d^	52	Outpatient specialty clinics; lithium responders and non-responders	229	22.6 (7–25)	K-SADS-PL; SADS-L	22% BDI, II, NOS, SCZBD (0%) 32% MDD (3%) 20% adjustment (41%) 9% minor mood (2%) 23% anxiety (12%) 30% SUD (16%) 11% neuro dev (6%) 2% DBD (0%)	84% index episode depressive Mean onset 16 ± 4 years Latency 4 years to hypo/mania Mean onset hypo/mania 20 years 0 pre-pubertal hypo/mania Antecedent anxiety predicted 2.5-fold-risk major mood disorder
Wals et al. (2001); Mesman et al. (2013)	The Nether-lands	Fixed^b^	12	Baseline, 1–5, 12-years (4)	23	93	75% BDI 25% BDII	60	72% patient association 28% outpatient clinic	108	28.0 (22–33)	K-SADS-PL; SCID	12% BDI, II, SCZBD 17% MDD 4% adjustment 29% minor mood 25% anxiety 23% SUD 5% ADHD, 7% DBD	88% index episode depressive Mean onset 15 ± 5 years Latency 5 years to hypo/mania Median onset hypomania 17 years Median onset mania 20 years 0 pre-pubertal hypo/mania
Egeland et al. (2003, 2012)	USA	Fixed	16	Annually (16)	0	15	100% BDI	43	Genetic linkage research; Amish community	115	59% within ‘window of risk’ period (13–29)	CARE - interview	7% BDI (1%), 39% Risk rating (16%)	Median onset hypo/mania 18 years 0 pre-pubertal hypo/mania All preceded by anxiety/dep 0 evidence of SMD in childhood 0 DBD, ADHD, irritability Episodic childhood internalizing-symptoms Shift in adolescence to externalizing-symptoms
Birmaher et al. (2009); Axelson et al. (2015)	USA	Fixed	Mean 6.8	Mean 2.5 years (mean# interviews 2.7)	9	236	72% BDI 28% BDII	81	53% advertisement 31% adult BD studies 16% outpatient clinics	356	18.1 (± 4.8)	K-SADS-PL; SCID	8% BD (1%), 11% BDNOS (1%) 19% MDD (14%), 10% Dep NOS (7%) 40% anxiety (22%) 20% SUD (10%) 31% ADHD (18%), 27% DBD (15%) 35% ODD/CD (19%)	Mean onset hypo/mania 13 years Mean onset MDD 14 years 33% index hypo/mania < 10 years 53% onset hypo/mania < 12 years
Nurnberger et al. (2011)	USA	Fixed	2–3	Baseline, 1–2-years (3)	No info	88 proband^c^	88.5% BDI 7% BDII 4.5% SCZBD	No info	Outpatient clinic; research studies	141	17 (12–21 baseline)	K-SADS-BP; 65% by telephone	9% BDI, II, NOS (0%) 16% MDD (4%) 18% SUD (9%) 8% ADHD (6%), 11% DBD (8%)	Median onset hypomania 16 years Median onset mania 13 years 5% of BD had onset ≤ 12 years Antecedent anxiety predicted a 2.5-fold-risk major mood disorder Antecedent externalising disorders predicted 3.5-fold-risk major mood disorder
Vandeleur et al. (2014); Preisig et al. (2016)	Switzer-land	Dynamic	Mean 10.6	Every 3 years (mean # interviews 3.3)	33	81	65.5% BDI 12.5% BDII 22% SCZBD	58	Inpatient and outpatient clinics	145	21.1 (16–28)	K-SADS-E	12% BDI, II (4%) 17% BPS (5%) 37% MDD (38%) 44% anxiety (38%) 28% SUD (19%) 17% ADHD (13%) 15% ODD (13%)	Mean onset hypo/mania 16 years (median = 15 years) Mean onset MDD 13 years (median = 13 years) 61% index episode depressive 19.4% onset hypo/mania < 12 years

*ADHD* attention deficit (hyperactivity) disorder, *BD* bipolar disorder, *BD*-*NOS* bipolar disorder not otherwise specified, *BD I* bipolar I disorder, *BD II* bipolar-II disorder, *BPS* bipolar spectrum disorder, *CD* conduct disorder, *DBD* disruptive behavioral disorder, *Dep*-*NOS* depression not otherwise specified, *MDD* major depressive disorder, *Neuro*-*dev* neurodevelopmental disorder, *ODD* oppositional defiant disorder, *SCZBD* schizoaffective disorder bipolar type, *SUD* substance use disorder

^a^ *Dynamic* continuous enrolment and follow-up from entry

^b^ *Fixed* enrolment at time 1 and then follow-up

^c^ *Proband* for 81% of offspring the proband was the parent

^d^ *RMDD* recurrent major depressive disorder in 1st degree relatives of BD probands

## Results and discussion

### Emerging bipolar disorder

Bipolar disorder runs in families and a confirmed history in a first-degree relative is the single most robust predictor of illness risk. Based on data from published family studies and a meta-analysis, a first degree relative of a bipolar patient has an estimated eight- to tenfold lifetime risk of developing bipolar disorder and a two to threefold lifetime risk of developing a depressive disorder compared to the general population (Duffy et al. 2000; Wilde et al. 2014). However, recent evidence from two large family studies found an increase of major depressive disorder only among adult family members of bipolar-II but not bipolar-I probands, suggesting the independence of familial aggregation of bipolar-I and major depressive disorder (Vandeleur et al. 2014; Merikangas et al. 2014). The discrepant results of these recent compared to earlier family studies is most likely explained by differential methodological approaches—that is a mixture of probands with both bipolar-I and bipolar-II disorders, as well as the use of RDC criteria and different diagnostic measures. Depressive disorders that share a bipolar diathesis (i.e., in relatives of bipolar probands) or represent the emerging course (i.e., in subjects that later develop bipolar disorder) have been described as having an early adolescent onset, a highly recurrent course, and not uncommonly include psychotic features, activated symptoms and evidence of paradoxical worsening on antidepressants (Strober and Carlson 1982; O’Donovan et al. 2008).

There are a number of risk modifiers that can be used to individualize the estimated risk of developing bipolar disorder for individuals from specific families. For example, high penetrance across multiple generations, an earlier age of onset in the proband bipolar patient, and assortative mating all increase the risk estimate (Duffy et al. 2000). Additionally, early adversity in the form of abuse, neglect, increased exposure to active parental illness, and attachment difficulties have been associated with an increased risk and an earlier age of onset of bipolar disorder, possibly through epigenetic mechanisms (Goodday et al. 2015; Duffy et al. 2016; Doucette et al. 2016; Petronis 2010; Post et al. 2016; Bagot and Meaney 2010).

Given the high estimated heritability of bipolar disorder (Smoller and Finn 2003), children of bipolar parents are an important and informative high-risk population. Prospective study of the emerging clinical course in children of parents with well-characterized bipolar disorder, alongside assessment of other important risk factors, is the best way to disentangle primary illness development from burden of illness effects. There are now a number of published international prospective longitudinal offspring studies (Table 1) (Duffy et al. 2014; Egeland et al. 2012; Mesman et al. 2013; Birmaher et al. 2009; Preisig et al. 2016; Nurnberger et al. 2011). Each study varies in terms of recruitment and assessment methods, yielding differences in risk modifiers such as the age of onset and comorbidity in the proband parents, degree of assortative mating, proportion of intact families, and level of education and income of the family. The nature of the comparator (control) group also differs across high-risk studies.

Nonetheless, there are several key findings regarding the children at confirmed familial risk that can be summarized as follows: (i) anxiety and sleep disorders in childhood increase the risk of subsequent major mood disorders 2.5- to 3-fold compared to high-risk offspring without these antecedents (Nurnberger et al. 2011; Duffy et al. 2013); (ii) ADHD and behavioural disorders are not consistently elevated in high-risk offspring after adjustment for family factors (socioeconomic status, cultural context, ADHD and other comorbid psychiatric disorders in bipolar and other parents) and bipolar subtype (psychotic spectrum illness) (Egeland et al. 2012; Birmaher et al. 2009; Nurnberger et al. 2011; Duffy 2012; Axelson et al. 2015); (iii) increased exposure in childhood to active parental illness (Goodday et al. 2015) and earlier age of onset of parental bipolar disorder (Preisig et al. 2016) appear to increase the risk of psychopathology in high-risk offspring.

Several offspring studies, but not all (Axelson et al. 2015), confirm the prominence of depressive mood disorders early in the course of emergent bipolar disorder, typically manifesting at the syndrome level in early to mid-adolescence (Duffy et al. 2009, 2014; Mesman et al. 2013; Nurnberger et al. 2011). To wit, the Dutch, multisite US, and Canadian offspring studies reported that in the vast majority of cases, the initial major mood episode in those offspring who developed bipolar disorder was depressive in polarity (Duffy et al. 2014; Mesman et al. 2013; Nurnberger et al. 2011). Interestingly, despite repeated prospective direct assessment of children at confirmed familial risk, these studies have almost uniformly found no support for the proposed pre-pubertal or very early onset bipolar disorder subtype (Duffy et al. 2014; Egeland et al. 2012; Mesman et al. 2013). For example, the mean age of onset of the first diagnosable activated episode across studies at last report ranged from 13 to 20 years (Duffy et al. 2014; Mesman et al. 2013; Nurnberger et al. 2011). The BIOS study stands apart somewhat from these other published studies reporting that, in those offspring who developed BD, 50% had mania prior to age 12 (compared to 0% in other studies) and 50% debuted with a depressive episode (compared to over 80% in other studies) (Axelson et al. 2015). It is possible that these differences reflect differences in study populations (Birmaher et al. 2009)—BIOS families had significantly lower SES, higher rates of comorbidity in the BD parent, and more assortative mating as indicated by high rates of psychiatric illness in the other (non-bipolar) parent compared to the Canadian, Dutch and Amish studies (Duffy et al. 2014; Egeland et al. 2012; Mesman et al. 2013). The Swiss study found the mean age of onset of the first diagnosable activated episode was age 16 (SD 5.1 years), of which 61% debuted with a depressive episode. Six cases (19.4%) of mania/hypomania were found prior to age 12, but 5 out of these 6 children had experienced early trauma just before the reported onset of mania/hypomania. The mean age of onset of the first activated manic/hypomanic episode in the remaining offspring was 17.5 (SD 4.3) years. With longer follow-up, the mean age of onset of first activated episode will likely increase further as new onsets continue to occur and as evidenced in the updated reports from the Dutch and Canadian studies (Duffy et al. 2014; Mesman et al. 2013).

**Fig. 1:**
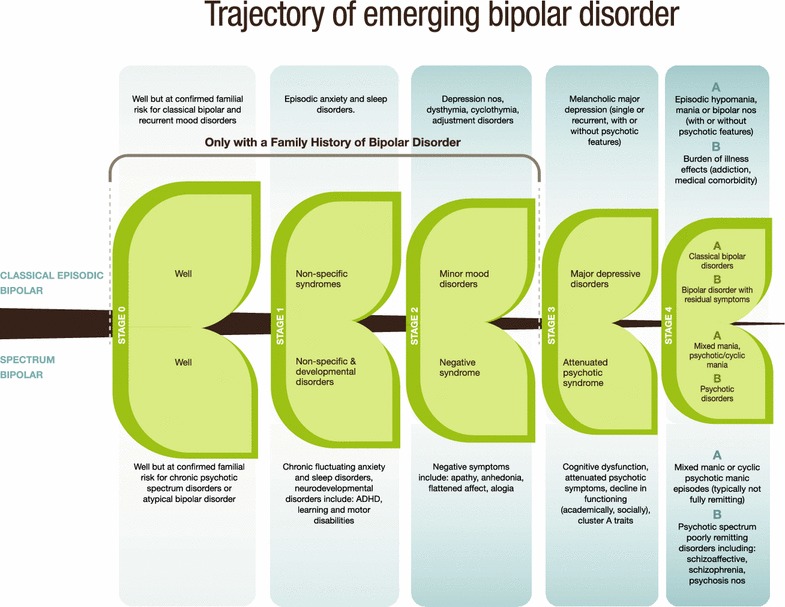
The trajectory of emerging bipolar disorder in two subtypes of high-risk offspring

A preliminary analysis of the trajectory of emerging bipolar disorder based on the Canadian Flourish high-risk offspring cohort was consistent with a progressive sequence of psychopathology over development (see Fig. 1 reproduced with permission (Duffy 2014). Further evidence supported that bipolar disorder varies in presentation within families and in course and treatment response between families, highlighting important aspects of phenotypic and etiologic heterogeneity. The best-fit model supported a shift from non-specific childhood antecedent syndromes (i.e., sleep problems, anxiety, and in the lithium non-responsive bipolar subtype, neurodevelopmental disorders) to internalizing symptoms under stress (i.e., sensitivity), to depressive disorders and finally on to the manifestation of the first activated episode.

This conceptual model of the trajectory of emerging bipolar disorder is consistent with several of the main observations among the high-risk studies, namely that: (i) bipolar disorder does not typically begin with a manic episode and is often presaged by childhood antecedent conditions and years of depressive episodes with more proximal subsyndromal hypomanic symptoms; (ii) childhood sleep and internalizing disorders likely have different significance in children with a confirmed familial risk of bipolar disorder compared to children without this risk; (iii) depressive episodes in adolescents at confirmed familial risk of bipolar disorder may represent emerging bipolar disorder or at least an increased likelihood of a bipolar predisposition; (iv) manic-like presentations in very young children without a confirmed family history of bipolar disorder may index a set of disorders or problems unrelated to bipolar disorder.

Findings from a recent meta-analysis indicate that there is substantive heterogeneity in prodromal symptom profiles and duration preceding an index manic episode (Van Meter et al. 2016). All but one of the studies included in this meta-analysis relied on retrospective data, often based on structured self-report symptom rating scales. Further, recall was over variable periods of time and often in patients whose most recent episode was manic. All of these methodological aspects are potential sources of bias. These limitations notwithstanding, common prodromal or proximal symptoms (> 50%) included excessive energy and goal directedness, talkativeness and pressured speech, elated mood, insomnia, depressed mood, diminished ability to think, and academic and work difficulties (Van Meter et al. 2016).

The Amish high-risk offspring study was the first to report that sensitivity by nature (i.e., stress reactivity) and episodic internalizing anxiety and depressive symptoms shifted into episodic activated symptom clusters over development and more proximal to the onset of bipolar disorder (Egeland et al. 2003, 2012). The episodic recurrent nature of these symptoms, combined with the confirmed familial risk of bipolar disorder in multiple generations, increases the likelihood that these otherwise non-specific manifestations reflect the bipolar diathesis.

Recently, the BIOS study showed that parent-reported internalizing symptoms and child-reported affective lability predicted onset of the first manic episode; the latter in part through an association with more proximal manic symptoms (Hafeman et al. 2016). The Flourish Canadian study observed that while the median age of onset of clinically assessed hypomanic symptoms in that offspring cohort was 16.4 years, the range was large (between 6 and 30 years). On average, these clinically significant hypomanic symptom onset prior but proximal to the index depressive episode (median onset age 16.8 years), and several years before the onset of the first diagnosable hypo/manic episode (median age of onset 18.4, bipolar I, and 21, bipolar-II) (Goodday et al. 2015). A recent report also found that high-risk offspring of bipolar parents had more severe depressive episodes and were more likely to experience mixed (activated) symptoms compared to low-risk depressed controls (Diler et al. 2017).

Cognitive function has been a focus of studies aimed at predicting risk and describing the early course of psychotic spectrum disorders, including bipolar disorder. Evidence from prospective studies, reviews, and a meta-analysis support distinctly different trajectories of cognitive function in schizophrenia and bipolar disorder over the emerging course of illness onset (Lewandowski et al. 2011; Trotta et al. 2015). Specifically, lower general intelligence and poorer school performance is evident pre-morbidly in children who later develop schizophrenia (MacCabe and Murray 2004; MacCabe et al. 2010). Further, at an early age, children who develop psychosis have evidence of pre-morbid cognitive deficits in verbal learning, working memory, attention, processing speed and executive function, along with “soft” neurological deficits, including problems with coordination and fine motor control (Owens and Johnstone 2006; Cannon et al. 2002). School performance, social functioning and cognitive deficits worsen through the school years leading up to the first psychotic break (Lewandowski et al. 2011). The trajectory of premorbid abnormal and deteriorating cognition and function across a number of domains, taken together with childhood neurological abnormalities, are consistent with a neurodevelopmental hypothesis of schizophrenia (Owens and Johnstone 2006; Clarke et al. 2012).

In contrast, based on prospective studies, the academic performance, cognitive function, and neurodevelopmental history of children who go on to develop bipolar disorder is comparable to that of the general population (Lewandowski et al. 2011; Trotta et al. 2015). Children who develop bipolar disorder do not manifest the same degree of lower and declining intellectual functioning or school performance as seen in children who develop schizophrenia (MacCabe et al. 2010; Cannon et al. 2002; Murray et al. 2004). Rather than reflecting the illness predisposition, cognitive deficits in bipolar disorder appear to largely manifest after illness onset (Lewandowski et al. 2011) and relate to aspects of the nature of the illness course, such as the number and polarity of mood episodes, polypharmacy, psychosis, antipsychotic treatment, and medical comorbidity (Berk et al. 2011; Hajek et al. 2013a, b; Calkin et al. 2015). However, it should be noted that studies have typically not considered heterogeneity of bipolar illness. Specifically, there is evidence to suggest that the psychotic spectrum bipolar subtype, as indexed by non-response to lithium prophylaxis, is characterized by learning disabilities, academic problems and ADHD in childhood—a point of overlap with the psychotic illness trajectory and differentiation from the early developmental history of those who develop classic lithium-responsive manic-depressive illness (Duffy 2012, 2015).

Alongside the evolution of clinical symptoms and syndromes, there is accruing evidence of the importance of psychological risk processes in the onset of bipolar disorder. These psychological risk processes in part overlap with those predicting unipolar depression in that children at genetic risk for bipolar disorder have unstable self-esteem and abnormal styles of self-regulation including excessive rumination (Jones et al. 2006a; Pavlickova et al. 2014). Additional psychological targets are suggested by studies showing that young people with hypomanic personality traits show abnormal reward responsivity, (Meyer et al. 1999) impulsiveness, (Mason et al. 2012) and abnormal interpretation of activated internal states, (Jones et al. 2006b) and that young people with abnormal sensitivity to reward and goal-striving are at elevated risk of developing BD symptoms (Alloy et al. 2012). It is possible that different psychological risk processes are important at different stages in the emerging clinical trajectory of specific subtypes of bipolar disorder and could be important intervention targets (Duffy et al. 2016).

## Conclusions

There is evidence from a number of longitudinal high-risk offspring studies that the index manic or hypomanic episode typically manifests in mid-late adolescence and early adulthood. Despite the fact that DSM considers the activated episode to be the cardinal indicator of bipolar illness, prospective high-risk studies show that activated episodes are often preceded by years of clinically significant psychopathology at both the syndrome and symptom levels. Internalizing symptoms, emotional sensitivity and sleep and anxiety disorders in childhood, followed by depressive disorders and more proximal prodromal hypomanic symptoms manifest prior to the full-threshold hypo/manic episode. Antecedent symptoms and syndromes may have a different predictive meaning in children at confirmed familial risk of bipolar disorder compared to children without this risk. Further, there is accumulating evidence for the importance of rumination, self-appraisal processes, impulsivity, and reward sensitivity in the development of bipolar disorder and these psychological risk processes may have different importance at different stages of the bipolar illness trajectory. In contrast to psychotic illnesses, cognitive deficits in bipolar disorder emerge after full-blown illness onset and are likely more related to mixed burden of illness effects, emphasizing the importance of accurate early identification and effective early intervention.

### Clinical implications

The observations from prospective high-risk studies can inform prevention and early intervention strategies at both the program and individual patient levels. While there are some practical and conceptual reasons for separating adult and youth psychiatric services, it is clear that this separation is detrimental when it comes to youth from families with a high risk of developing major psychiatric disorders. Therefore, closer collaboration between child and adult psychiatric services and other medical programs seems crucial—both to identify children at risk given a confirmed family history and to work to improve the mental health of affected parents with children (this may be especially important for bipolar mothers with children). In addition, further development of programs designed specifically targeting symptomatic transitional aged youth (15–25 years) is a necessity. These programs should offer engaging and accessible psychoeducation and support programs to mitigate risk factors and bolster resilience including healthy coping with stress, mood regulation, and avoidance of substance use. These community-based programs should have direct access to subspecialty early intervention programs that provide timely expert assessment and evidence-based care to those with emergent illness, especially those with a confirmed familial risk of major psychiatric illness and/or suicide.

At an individual level, to more accurately predict risk, prognosis, and treatment response in young people, who often have limited and seemingly non-specific clinical presentations, the current approach to diagnosis should be supplemented with other predictive information including: penetrance and nature of psychiatric illnesses in relatives, age on onset of illness in first-degree relatives, developmental history, psychosocial risk exposures, and the clinical course of emerging psychopathology. The outcome of this more comprehensive approach will earlier illness identification, more effective and better-tolerated interventions, and improved long-term outcomes for patients.

### Research gaps and next directions

Longitudinal studies of children of well-characterized bipolar parents and their adult relatives will advance understanding of pathophysiological mechanisms, translating predisposition to illness onset and identifying more homogeneous bipolar subtypes. Prospective offspring studies make possible the mapping of changes in multilevel indicators of illness risk from pre-morbid states to prodromes and finally to illness onset. Early risk exposures (trauma, abuse, exposure to parental active illness), psychological risk processes, and biological markers can be mapped to the emerging clinical course through parallel studies. Understanding the nature of the illnesses segregating in family members and the family environment is also an important contributor to risk and outcomes in the children. These studies can also advance understanding of complex pathways leading to complications and comorbidities of bipolar disorder such as substance use, suicide and cardiovascular illness.

To advance, future research will need to consider the effect of heterogeneity on outcomes. As currently defined, bipolar disorder is a broad diagnostic category comprised of different illness subtypes with varying pathophysiology as evidenced by differences in clinical course, family history, treatment response, genetic, and neurobiological findings (etiologic heterogeneity) (Manchia et al. 2013; Angst et al. 2004). One type of bipolar disorder appears related to psychotic illness with early adolescent onset of psychotic manic or mixed episodes, male preponderance, neurodevelopmental comorbidity, poor quality of remission, family history of psychotic or chronic mood disorders and failure to fully respond to lithium prophylaxis (Grof et al. 2009; Alda 2004). By contrast, another type of bipolar disorder is consistent with the Kraepelin description of classical manic-depressive illness characterized by a highly recurrent course, predominance of depressive episodes, good quality of spontaneous remission, equal sex ratio, family history of episodic mood disorders, and an excellent response to lithium prophylaxis (Grof et al. 2009; Alda 2004).

There is also evidence of phenotypic heterogeneity in that the bipolar trait presents differently in affected relatives within the same families, who presumably share the same illness subtype or trait. Furthermore, the spectrum of illness (phenotypic spectrum) differs between subtypes. For example, relatives of patients with a classical lithium-responsive manic-depressive illness manifest high rates of episodic major depression and bipolar I and II disorders, while relatives of patients with psychotic spectrum bipolar disorder have elevated rates of chronic illnesses including neurodevelopmental disorders (autism, learning disabilities, ADHD), schizoaffective illness, chronic depression, and psychosis (Duffy and Grof 2001; Rice et al. 1987).

More recently, early intervention research concerning psychotic disorders has focused on studying heterogeneous populations of youth at clinical high-risk of developing psychosis. While this approach has been very helpful in identifying individuals manifesting high-risk mental states warranting intensive surveillance and clinical intervention, the approach is not likely robust enough to inform the identification and validation of biomarkers or to understand the translation of genetic predisposition into illness onset. To advance our understanding of bipolar disorder onset, it will be necessary to start from parents with well-characterized bipolar illness that can be organized into more homogeneous subtypes. Refined illness phenotypes have a higher likelihood of mapping to changes in underlying genetic and neurobiological markers and thereby identifying specific intervention targets. Whilst findings may not be directly generalizable to heterogeneous at risk and clinical populations, it is a necessary approach to advance our understanding of the pathophysiology driving emerging illness. Similar approaches have been very productive in differentiating illness subtypes and identifying susceptibility genes and biological markers associated with illness progression in other areas such as autism (Loth et al. 2017), dementia (Meeter et al. 2017), and cancer research (Zabor and Begg 2017).
